# Automated segmentation and feature discovery of age-related macular degeneration and Stargardt disease via self-attended neural networks

**DOI:** 10.1038/s41598-022-18785-6

**Published:** 2022-08-26

**Authors:** Ziyuan Wang, Srinivas Reddy Sadda, Aaron Lee, Zhihong Jewel Hu

**Affiliations:** 1 Doheny Eye Institute, 150 N Orange Grove Blvd, Pasadena, 91103 USA; 2 The University of California, Los Angeles, CA 90095 USA; 3 The University of Washington, Seattle, WA 98195 USA

**Keywords:** Computational biology and bioinformatics, Diseases, Engineering

## Abstract

Age-related macular degeneration (AMD) and Stargardt disease are the leading causes of blindness for the elderly and young adults respectively. Geographic atrophy (GA) of AMD and Stargardt atrophy are their end-stage outcomes. Efficient methods for segmentation and quantification of these atrophic lesions are critical for clinical research. In this study, we developed a deep convolutional neural network (CNN) with a trainable self-attended mechanism for accurate GA and Stargardt atrophy segmentation. Compared with traditional post-hoc attention mechanisms which can only visualize CNN features, our self-attended mechanism is embedded in a fully convolutional network and directly involved in training the CNN to actively attend key features for enhanced algorithm performance. We applied the self-attended CNN on the segmentation of AMD and Stargardt atrophic lesions on fundus autofluorescence (FAF) images. Compared with a preexisting regular fully convolutional network (the U-Net), our self-attended CNN achieved 10.6% higher Dice coefficient and 17% higher IoU (intersection over union) for AMD GA segmentation, and a 22% higher Dice coefficient and a 32% higher IoU for Stargardt atrophy segmentation. With longitudinal image data having over a longer time, the developed self-attended mechanism can also be applied on the visual discovery of early AMD and Stargardt features.

## Introduction

Age-related macular degeneration (AMD) and Stargardt macular dystrophy are two ophthalmic diseases affecting the retina, causing degeneration of the macula. AMD is the leading cause of vision loss in older adults, impacting roughly 200 million people around the world^1–4^. Stargardt macular dystrophy is also called Stargardt disease or juvenile macular degeneration (JMD) which has been shown to be the leading cause of inherited vision loss in young adults/children and can worsen to cause legal blindness^5,6^. Geographic atrophy (GA), a form of late-stage AMD, manifests itself as a region of loss of the photoreceptors and retinal pigment epithelium (RPE), while early-stage AMD is largely asymptomatic^2^. Similarly, late-stage Stargardt disease can also manifest as region of atrophy, although distinct from that of AMD^5,6^. Fundus autofluorescence (FAF) imaging is a non-invasive, in-vivo technique which provides a 2-D mapping of the metabolically occurring fluorophores of the ocular fundus^7^. As atrophy ensues, there is a loss of RPE and hence, a loss of fluorophores in the lipofuscin which is normally located within the RPE cells. The loss of the RPE and corresponding fluorescence, creates well-demarcated regions of hypo-fluorescence which are representative of the region of atrophy. Due to the high image contrast for defining these regions of photoreceptor and RPE atrophy, FAF images have been widely used for the diagnosis and analysis of AMD GA and Stargardt atrophy in clinics.

As AMD GA and Stargardt atrophy are the end-stage outcomes of these two retinal diseases respectively, efficient and accurate methods for the segmentation and quantification of these atrophic regions in FAF images are critical for clinical research studies attempting to better understand the disease progression. Furthermore, early detection and knowledge of key image features or biomarkers indicative of which eyes are likely to progress most rapidly is of importance in identifying which patients may benefit the most from early intervention with novel therapies before irreversible vision loss has occurred. Recognizing these biomarkers may also be of value in enhancing our understanding of disease pathophysiology.

Traditionally, atrophy segmentation for AMD and Stargardt disease in clinical research studies have been completed manually or at best semi-automatically by trained graders^7–11^. Likewise, AMD and Stargardt biomarkers have traditionally been identified by means which involve the direct input of clinicians and scientists. Manual involvement of these processes, however, is tedious and time-consuming. Automated approaches have become the preferred technique for image segmentation and analysis. We reported a traditional machine learning approach with $k$-nearest neighbor ($k$-NN) classifier for automated AMD GA segmentation^12^. It utilized hand-crafted filters to extract image features, therefore requiring expertise, and was difficult to generalize to the large variations of ophthalmic image data. In contrast, deep learning algorithms automatically learn interesting image features for optimal segmentation results and have the capability to be generalized. In parallel with the evolution of deep learning convolutional neural networks (CNNs) in recent years, much research has been done regarding the efficiency of CNNs in automated medical imaging analysis, particularly in segmentation tasks^13–33^. Earlier deep learning algorithms for semantic segmentation utilized deep CNNs with sliding windows and fully connected layers.

which are slow and less efficient^15^. A fully convolutional neural network architecture, the U-Net, has emerged as a state-of-the-art deep learning CNN approach for image segmentation in various eye-related assessment tasks including atrophic lesion segmentation^13,16–22^ for AMD^16–21^ and Stargardt disease^22^.

In recent years, efforts have been made to understand and visualize where neural networks focus their “attention” on images in different CNN layers, while paying less “attention” elsewhere. There are in general two major types of attention mechanisms related to CNNs, categorized as trainable or non-trainable (i.e., post-hoc attention). Early efforts in exploration of attention mechanisms were more focused on post-hoc attention, for example, the heatmap visualization approaches via multi-layered deconvolutional network (deconvnet)^30,31^ and via class activation mapping (CAM)^32,33^. Such post-hoc attention mechanisms helped visualize which parts in an image an already-trained CNN model deems important. Most recently, the focus has shifted to the exploration of trainable attention mechanisms which mimic how human eyes work^34,35^. Prior work has been done involving the trainable attention mechanism with CNNs for biomedical image analysis^34^. A trainable attention mechanism is supposed to help a CNN to attend key elements of images during training (as well as testing) and hence can help improve the CNN’s performance.

In this project, we developed an improved atrophic lesion segmentation system which embeds the trainable attention mechanism into the regular U-Net. Moreover, our training attention mechanism U-Net system generates soft-labels, which can provide more informative probabilistic predictions than deterministic hard labels. Hence, our neural network features an integrated soft-labeled self-attended CNN system, which does not need to train complex external parameters, and can efficiently suppress feature activation of the background regions and highlight the important foreground information with higher probability towards enhanced CNN performance.

To explore the potential applications and evaluate our self-attended neural networks, we performed several experiments. Firstly, we applied the self-attended neural networks for segmentation of AMD GA and Stargardt atrophic regions on baseline FAF images, against the corresponding manual ground truth based on baseline FAF images. Our previously developed regular U-Net is also applied on the same baseline datasets to compare the atrophy segmentation performance with the self-attended neural networks. Secondly, in an attempt to locate early features/biomarkers that indicate relevant information about the progression of atrophy in AMD and Stargardt disease, we applied the self-attended fully convolutional neural network on baseline FAF images, with their corresponding manually delineated ground truth depicting the development of the atrophic regions taken 12 months after the baseline imaging. Thirdly, visual heatmaps based on the trainable attention mechanism for the self-attended neural networks were generated. For comparison, visual heatmaps based on a post-hoc attention mechanism for the already trained regular U-Net model were also generated via the deconvnet.

## Methods

### Overview

**Figure 1:**
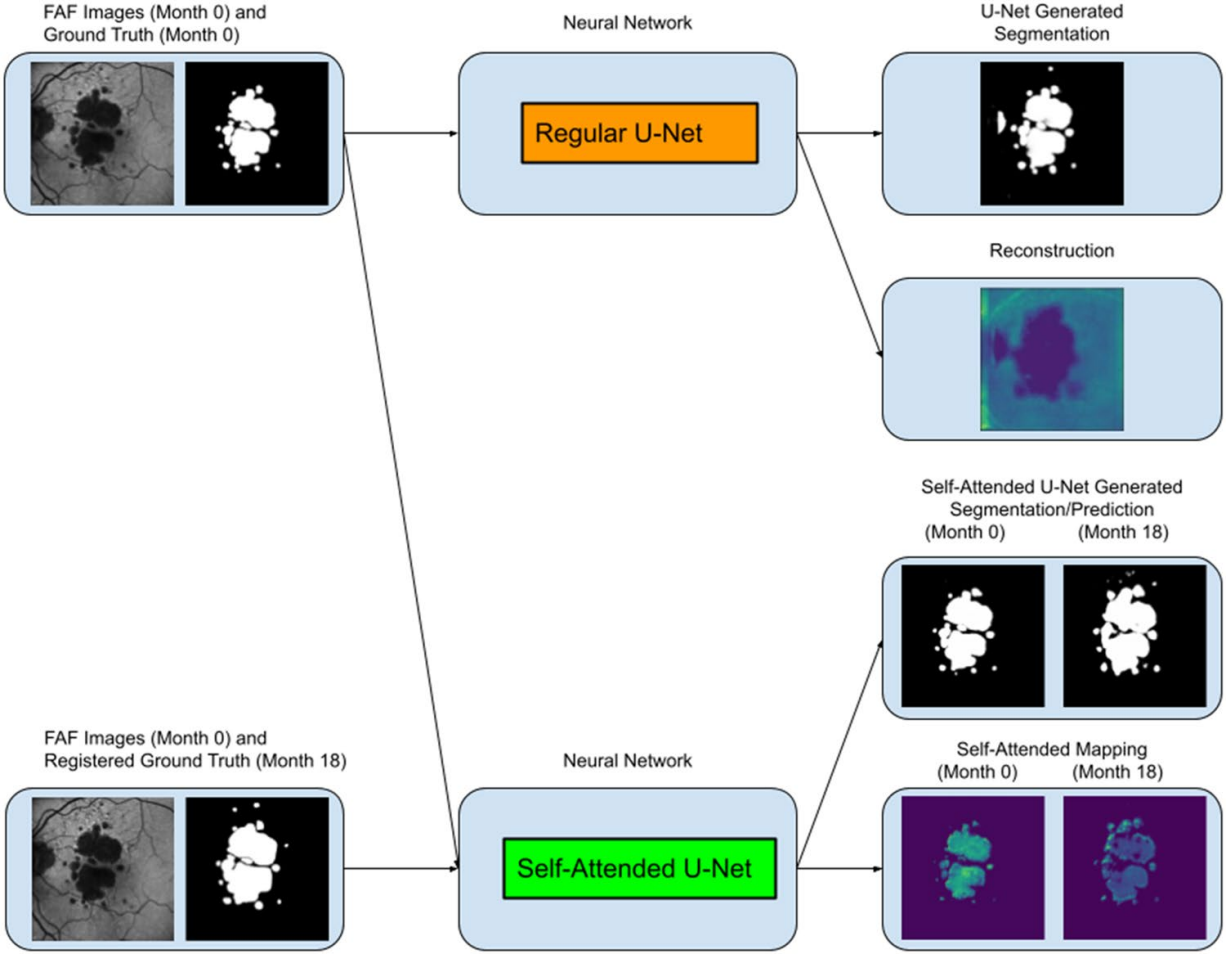
Overview of the entire atrophy segmentation and prediction system using regular U-Net and self-attended U-Net with AMD data as an example.

Figure 1 provides an overview of our AMD and Stargardt atrophy segmentation and prediction system using a regular U-Net and our soft-labeled self-attended U-Net, with AMD data as an example. The regular U-Net is only applied on baseline (Month 0) image data along with manual delineations for the comparison of atrophy segmentation with the self-attended U-Net. The self-attended U-Net is also applied on baseline (Month 0) image data along with registered manual delineations from follow-up (Month 12) images to predict the atrophy progressive growth. Furthermore, self-attended maps are obtained for the visualization of image features from the self-attended U-Net, and reconstruction maps are obtained for the visualization of the salient image features of the regular U-Net.

### Materials

#### Patients, images, and labelling

For Stargardt data, 206 eyes from 127 patients were included. For AMD data, 90 eyes from 90 patients (one eye per subject) were included. The inclusion criterion for age for Stargardt patients was at minimum 6 years or older, and for the AMD patients was 60 years or older. Both Stargardt and AMD patients needed to have well-demarcated area of atrophy in the absence of fluid. The images for both AMD and Stargardt disease were centered on macular regions. The field of view (FOV) for AMD was 30° and the FOV for Stargardt disease was 20°. In total, there were 90 AMD eyes with GA and 206 Stargardt eyes with Stargardt’s atrophy included in this institutional review board (IRB)—approved project. All the data used for the image segmentation for both AMD and Stargardt data were from baseline visits and for prediction with additional longitudinal data. More specifically, all the AMD and Stargardt eyes had FAF imaging (Spectralis HRA + OCT, Heidelberg Engineering) performed at baseline (labeled as Month 0), and then had follow-up FAF imaging done 12 months after (labeled as Month 12). The Stargardt images had an original size of 512 × 512 pixels, and the AMD images had an original size of 768 × 868 pixels. All images were de-identified prior to the application of the algorithms according to the Health and Insurance Portability and Accountability Act Safe Harbor. Individual patient’ age and sex information were not accessible for this study.

The GA regions on all the AMD FAF images and the Stargardt atrophy regions on all the Stargardt FAF images from both baseline and follow-up visits were manually delineated/labeled by certified reading center graders and were utilized as the ground truth. In the baseline atrophy segmentation algorithms’ training and testing for both the regular U-Net and the self-attended U-Net, the baseline images and corresponding manual labels are utilized. In the prediction algorithm’s training and testing of the self-attended U-Net for both AMD and Stargardt data, the original FAF images from the Month 12 visit were not used, only their manual labels (aligned with baseline images as described in “Longitudinal image and label alignment”) along with corresponding baseline images for ground truth for training and testing of the CNNs to predict progression of the diseases. Since the AMD FAF images included a black border on the bottom, we automatically detected and removed the black borders of these images (and cropped the black borders from the manually delineated ground truth) before algorithm training and testing, and then resized the images and manual labels to a constant size of 768 × 768 pixels. We used an eight-fold cross validation approach due to the relatively small size of the data set. The same 10 and 22 AMD and Stargardt eyes were used respectively as validation data. Eight rotating sets of 10 AMD eyes and 23 Stargardt eyes were used for testing, with 1 different set used for testing and the rest used for training for each fold.

#### Standard protocol approvals and patient consents

All methods were carried out in accordance with relevant guidelines and regulations. For AMD eyes, ethics review and institutional review board approval from the University of California—Los Angeles were obtained. For Stargardt eyes, the ethics reviews and institutional review board approvals were obtained from the local ethics committees of all the nine participating institutions, i.e., The Wilmer Eye Institute, Johns Hopkins University, Baltimore, Maryland (JHU); Greater Baltimore Medical Centre, Baltimore, Maryland (GBMC); Scheie Eye Institute, University of Philadelphia, Philadelphia, Pennsylvania (PENN); Retina Foundation of the Southwest, Dallas, Texas (RFSW); Moran Eye Centre, Salt Lake City, Utah (MEC); Cole Eye Institute, Cleveland Clinic, Cleveland, Ohio (CC); Moorfields Eye Hospital, London, UK (MEH, UK); Université de Paris 06, Institut national de la santé et de la recherche médicale, Paris, France (INSERM, France); and Eberhard-Karls University Eye Hospital, Tuebingen, Germany (EKU, Germany). Informed consent from all subjects and/or their legal guardian(s) for both study participation and publication of identifying images was obtained.

### Longitudinal image and label alignment

**Figure 2:**
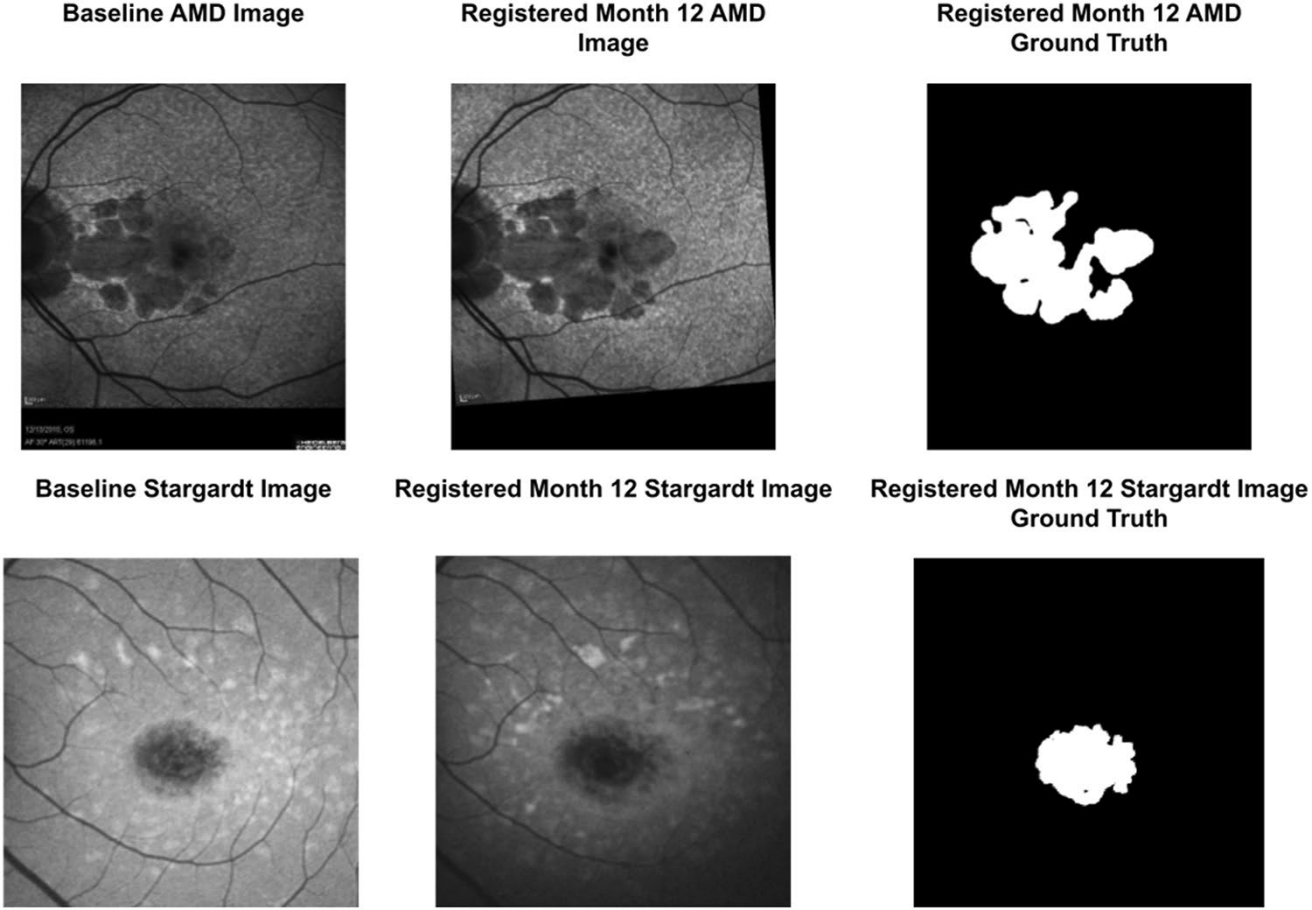
Illustration of longitudinal image and label alignment for AMD and Stargardt data. Note the hypo-fluorescence regions on the FAF images are AMD atrophic lesions (i.e., GA) (upper row) and Stargardt atrophic lesions (bottom row) respectively.

To predict the atrophy progression from AMD and Stargardt using the self-attended U-Net, we utilized the baseline FAF images and the corresponding manual labels of Month 12 for the algorithm training, which were longitudinally aligned. A feature-based image registration approach with rigid transformation which based on vessel branching, bifurcation, and crossover points was utilized to first align each Month 12 FAF image to its corresponding baseline image^36^. The same spatial transformation was then also applied on the Month 12 manual label image to obtain the registered ground truth for the training and testing of the self-attended U-Net for the prediction of the atrophy progression over time for both AMD and Stargardt. A set of longitudinal AMD and a set of Stargardt FAF image and ground truth registration results are demonstrated in Fig. 2.

### Self-attended deep CNNs

**Figure 3:**
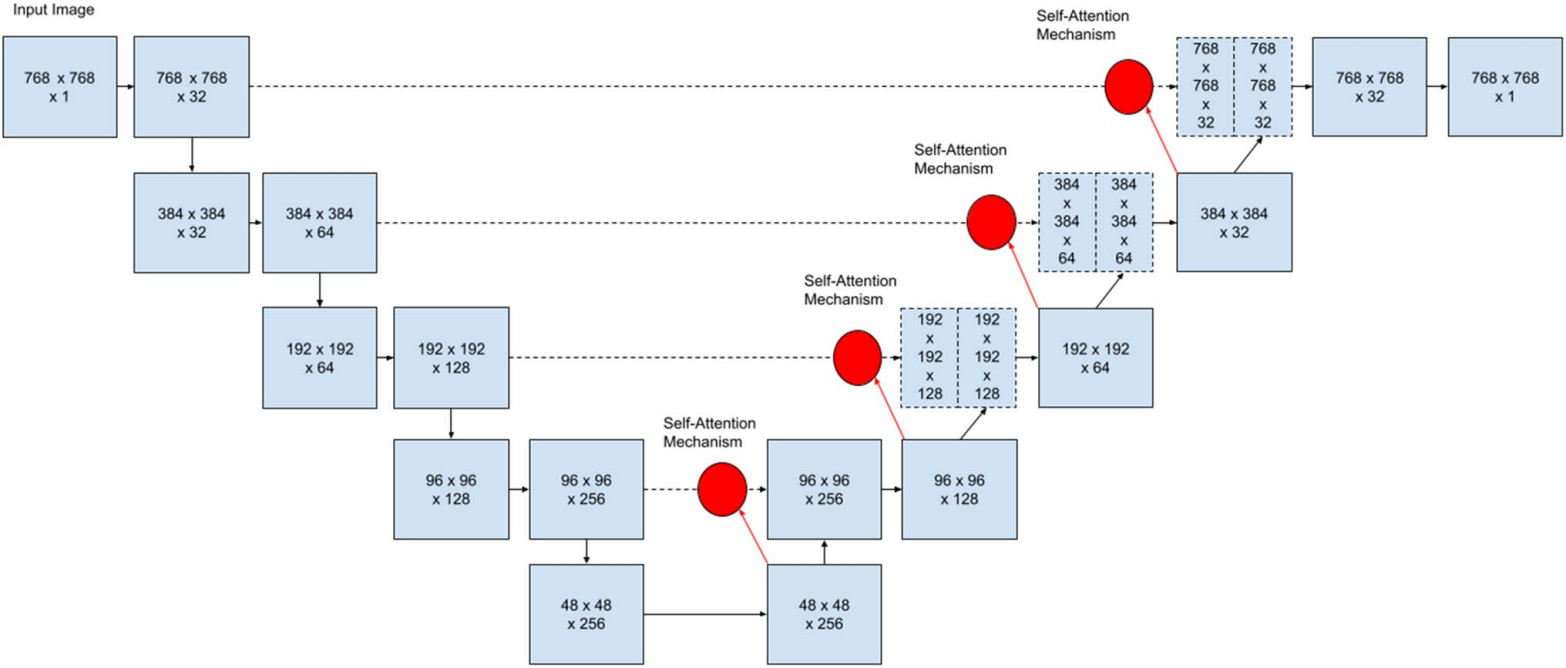
Illustration of the self-attended deep CNN mechanism/architecture.

We embed the self-attended mechanism directly on different deep CNN layers of the proven state-of-the-art U-Net. Such a CNN system is concise without the dependency of complicated external network components and parameters, and yet more effective and efficient than the regular U-Net. Figure 3 illustrates the self-attended deep CNN mechanism/architecture.

The U-Net is a fully convolutional neural network algorithm, without the need for fully connected layers. It includes a contracting auto-encoder path to capture context and a symmetric expanding auto-decoder path to enable precise localization. U-Net overcomes the bottleneck limitations of the classic auto-encoder by adding skip connections that concatenate the higher resolution features from the downsampling with the upsampled features for more efficient algorithm performance. For our self-attended U-Net, the output of the skip connections depends on the self-attention mechanisms. By embedding the self-attended mechanism in the regular U-Net, the self-attended mechanism highlights features to pass through the skip connections and, during the backward pass gradients originating form the background region are down weighted. The embedded self-attended mechanism progressively suppresses feature responses in regions that are irrelevant in the native CNN layers, allowing the neural network to focus on key details. As such, there is no need for training additional preceding image localization models to improve accuracy.

The output after the application of each self-attended mechanism is an element-wise combination of the input feature maps from the corresponding CNN layer of the U-Net, along with the self-attention coefficients of that layer. The feature maps ${x}^{l}$ for a given CNN layer are a standard part of U-Net and generated by two convolutions followed each by a rectified linear unit (ReLU). Self-attention coefficients *a*^*l*^_*i*_ ∈ [0, 1] are generated for each pixel *i* of the feature maps for each CNN layer *l* through the concatenation of the self-attended mechanism ${{s}_{i }^{l}\in {\mathbb{R}}}^{{F}_{s}}$ and connecting the input features ${x}_{i}^{l}{\in {\mathbb{R}}}^{{F}_{l}}$. ${F}_{s}$ and ${F}_{l}$ represent the numbers of self-attended mechanism signals and feature maps of each CNN layer. To concatenate them, both undergo linear transformations ${W}_{x}\in {\mathbb{R}}^{{F}_{l} x {F}_{int}}$, ${W}_{s}\in {\mathbb{R}}^{{F}_{s} x {F}_{int}}$ , and ${\psi }^{T}\in {\mathbb{R}}$^Fint×1^ to be mapped to an intermediate space with ${\mathbb{R}}^{{F}_{int}}$ dimensions.

1 $${a}_{i}^{l}= {\psi }^{T}({\sigma }_{1}\left({W}_{x}^{T}{x}_{i}^{l}+{W}_{s}^{T}{s}_{i}^{l}+{b}_{g}\right))+{b}_{\psi }$$

As a result, the self-attention coefficients ${\alpha }_{i}^{l}$ can be represented as:

2 $${\alpha }_{i}^{l}={\sigma }_{2}({a}_{i}^{l})$$

where ${b}_{g}\in {\mathbb{R}}$^Fint^ and ${b}_{\psi }\in {\mathbb{R}}$ are two bias terms, ${a}_{i}^{l}$ represents the combined input signal under a ReLU operation ${\sigma }_{1}$ and the linear transformation ${\psi }^{T}$, which is further sent to a sigmoid activation function ${\sigma }_{2}({x}_{i}^{l})= \frac{1}{\mathrm{exp}(-\frac{{x}_{i}^{l}}{T})}$.

The atrophy segmentation is a problem for two classes which classify each pixel on a FAF image to atrophy or non-atrophy. The sigmoid function converts the combined self-attention signals ${a}_{i}^{l}$, computed for each class, into a probability ${P(a,T)}_{i}^{l}$. In other words, a self-attention coefficient ${\alpha }_{i}^{l}$ is a probability

3 $${\alpha }_{i}^{l}= {P(a,T)}_{i}^{l} = \frac{1}{\mathrm{exp}(- \frac{{a}_{i}^{l}}{T})}$$

where $T$ is a temperature that is normally set to 1 for producing a harder label. Using a higher value for $T$ produces a softer probability distribution (soft label) over classes. A soft label indicates that the classification outcome of a member of a class is probability or likelihood encoded. The soft-label predictions are more informative about the predicted probability distribution of data points belonging to individual classes. In this study, $T$ was set to 1 for the ease of comparison with the regular U-Net.

### AMD GA and Stargardt atrophy segmentation, progressive prediction, and feature visualization

As mentioned above, we first applied the self-attended U-Net for the segmentation of atrophic lesions from AMD and Stargardt disease on baseline Month 0 FAF images against their corresponding baseline manual ground truth. Meanwhile, we also performed same experiments using our regular U-Net. Feature maps were obtained based on the self-attended mechanism as described above for the visualization of significant self-attended CNN signals. Additionally, image features for the regular U-Net were reconstructed and visualized via transposed convolutions replacing the standard convolutions by deconvnet as described in^30,31^. The reconstruction blocks operated in reverse, so that the inputs to the reconstruction blocks were first passed through the activations before the transposed convolutions. The inverse operation could place the maximal values kept after the forward max pooling back in the stored location and zero out remaining smaller values. Through recovering the inputs to each block in this way, we produced reconstructions of the notable signals for each CNN layer of the forward pass.

Furthermore, the self-attended U-Net was also applied to predict progression of AMD GA and Stargardt atrophy. As such, the baseline FAF images of AMD GA and Stargardt atrophy were trained with ground truth that was their corresponding manual segmentation from a follow-up visit 12 months after the baseline images were taken. The self-attended U-Net maps were also generated to attend the notable features for the atrophy progression after 12 months. The self-attended U-Net using baseline Month 0 images and manual ground truths gave us a comparison against the regular U-Net, and using baseline 0 images and the corresponding follow-up Month 12 manual ground truths can possibly reveal information about early features/biomarkers which predict atrophy progression after 12 months.

For the algorithm development, an open-source deep learning framework Keras, which provides a Python interface for neural networks and runs on top of TensorFlow, is utilized. A semiautomated software tool RegionFinder (Heidelberg Engineering) was used for the grading of both AMD and Stargardt atrophic lesions’ area on FAF images. More specifically, the images were first graded by a certified reading center grader using RegionFinder and then reviewed or adjusted by a senior grader. When there was discrepancy between the two graders, an adjudication was performed by a senior investigator.

### Algorithm performance evaluation

Generally, the performance of a CNN is greatly increased when the size of the dataset is sufficient and suffers when the data set is small. To overcome this problem with our relatively small data set, we performed an eightfold cross validation. For the models trained on FAF images containing AMD, each fold had ten test images, 70 training images, and ten validation images. While the ten selected test images were swapped with ten unique images from the set of training images on each fold, the validation images were kept the same. For the models trained on FAF images with Stargardt’s disease, each fold had 23 test images, 161 training images, and 22 validation images. This was done to keep the ratio of test, training, and validation images nearly the same between the models trained on AMD images and the models trained on Stargardt images. Likewise, the 23 test images were swapped out with 23 unique training images for each fold, while the validation images were kept constant. The same dataset (including training, validation, and testing) for every fold for both U-Net and self-attended U-net was used in this study. In Stargardt dataset, some patients had images for both eyes. To avoid bias in the testing for Stargardt data, for any two images of both eyes from a same patient, they were either both included in the training dataset or in the testing dataset but not included in training and testing separately.

Five metrics were used to measure the results: accuracy, sensitivity, specificity, Dice coefficient, and the intersection over union.

The accuracy measures the proportion of pixels on FAF images with correctly identified atrophy (TP, or true positive) and without atrophy (TN, or true negative), over the total of all identified atrophy and non-atrophy (including the false positives, FP, and false negatives, FN).

$$Accuracy=\frac{TP + TN}{TP+FP+TN+FN}$$

The sensitivity measures the amount of atrophy that is correctly identified, over all regions of atrophy. All regions of atrophy include those that were falsely identified as not having atrophy.

$$Sensitivity=\frac{TP}{TP+FN}$$

The specificity measures the amount of non-atrophy that was correctly identified, over all regions of non-atrophy. All regions of non-atrophy include those that were falsely identified as atrophy.

$$Specificity=\frac{TN}{TN+FP}$$

The Dice coefficient and intersection over union (IoU) both measure the spatial overlaps of an atrophic region A on an FAF image from manual delineation against the corresponding atrophy region B generated by algorithm segmentation.

$$Dice=\frac{2\left(A \cap B\right)}{A+B}$$

$$IoU=\frac{A \cap B}{A \cup B}$$

We performed four comparisons, i.e., the self-attended U-Net trained on baseline atrophic AMD FAF images and baseline manual delineation against a regular U-Net trained on baseline atrophic AMD FAF images and baseline manual delineation, the self-attended U-Net with baseline Stargardt images and baseline manual segmentation for ground truth compared to the regular U-Net trained on baseline Stargardt images and baseline ground truth, the self-attended U-Net trained on baseline AMD images and ground truth compared to the self-attended U-Net trained on baseline Stargardt images and baseline segmentation as ground truth, and the self-attended U-Net trained on baseline AMD images with 12-month progressed atrophy manual segmentation as ground truth compared to the baseline Stargardt images with 12-month progressed atrophy manual segmentation as ground truth, in percent differences for the above five evaluation metrics. Mann–Whitney test was also performed for each comparison for the analysis of statistically significant difference.

## Results

**Figure 4:**
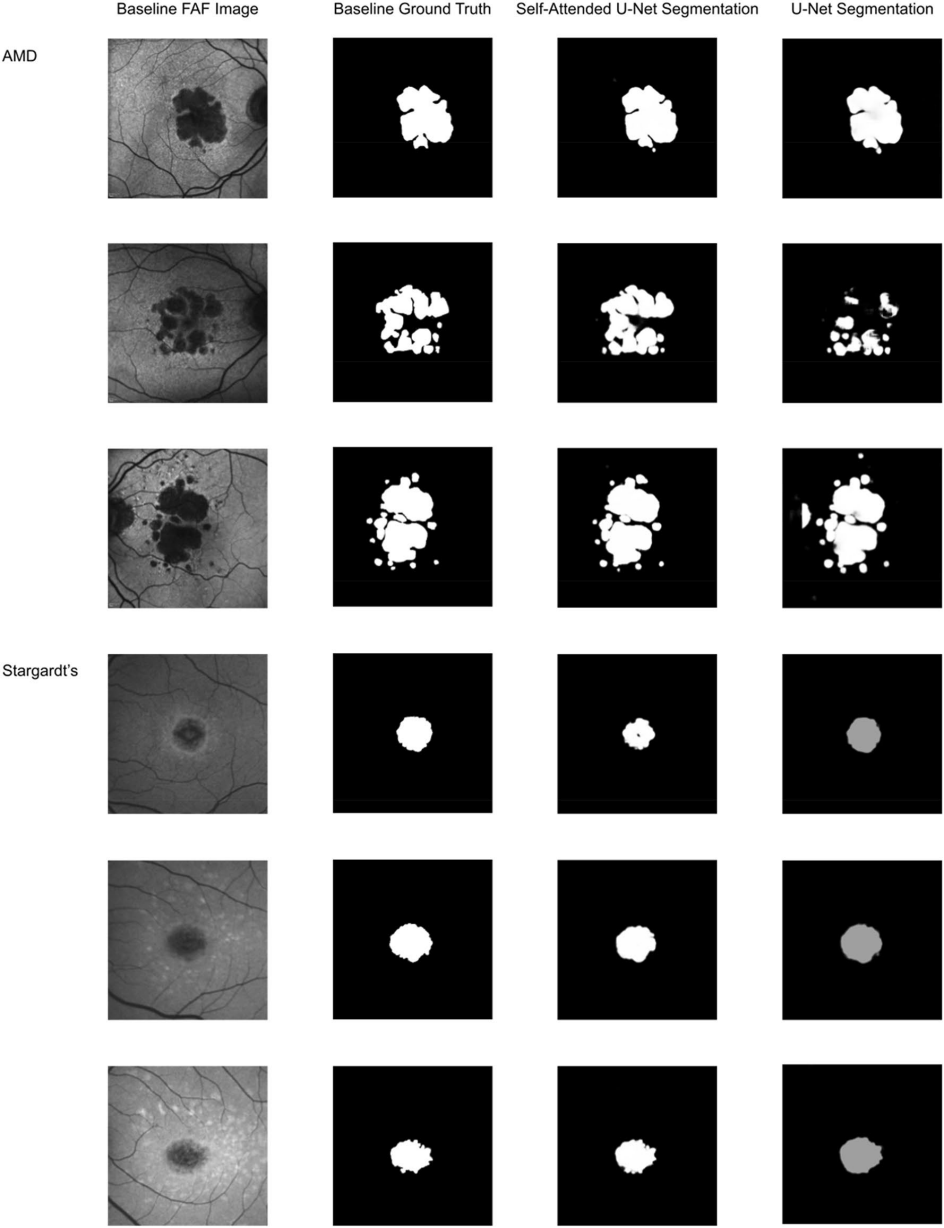
Illustration of atrophy segmentation results of the self-attended U-Net and the regular U-Net on baseline images with a representative performance.

**Figure 5:**
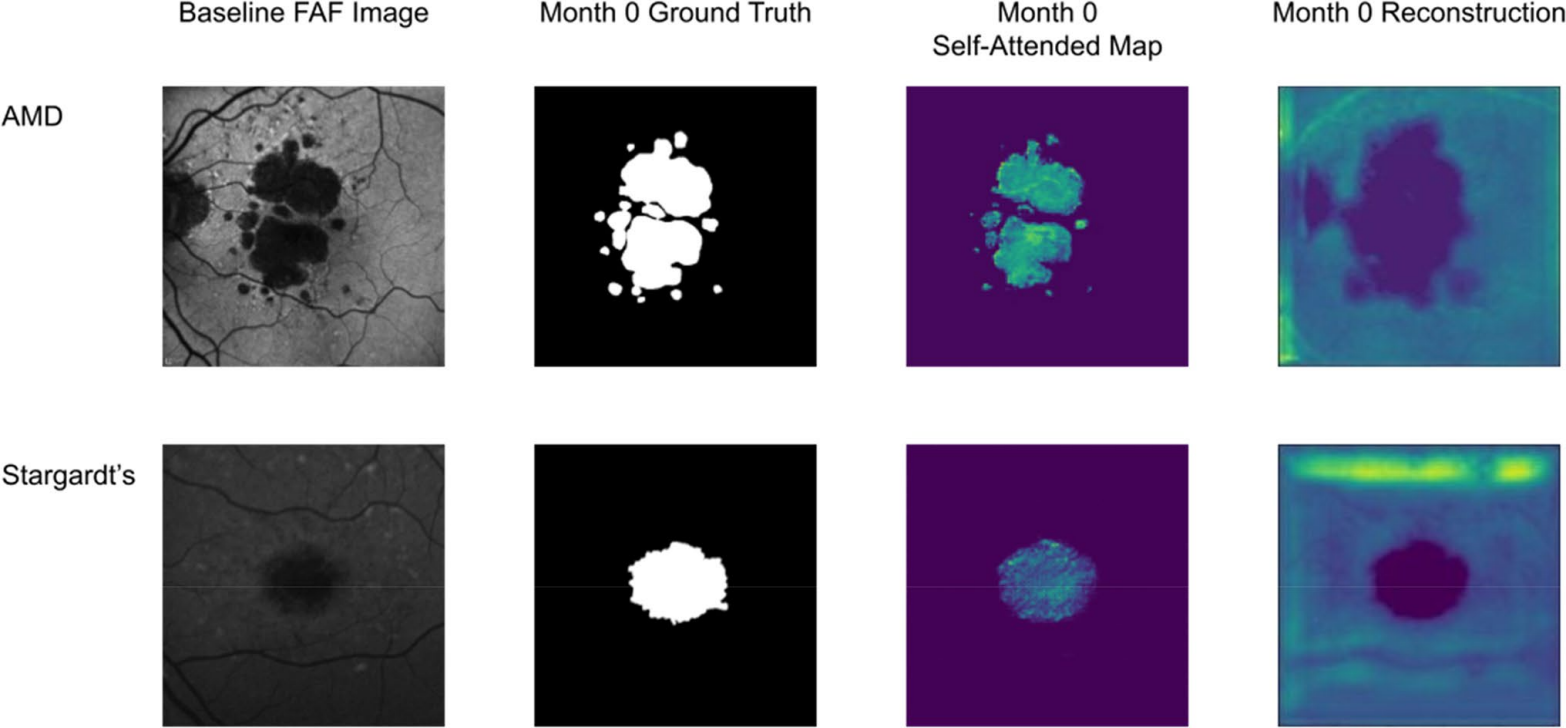
Illustration of the reconstruction and self-attended maps based on the baseline atrophy segmentation in the last downsampling layer.

**Figure 6:**
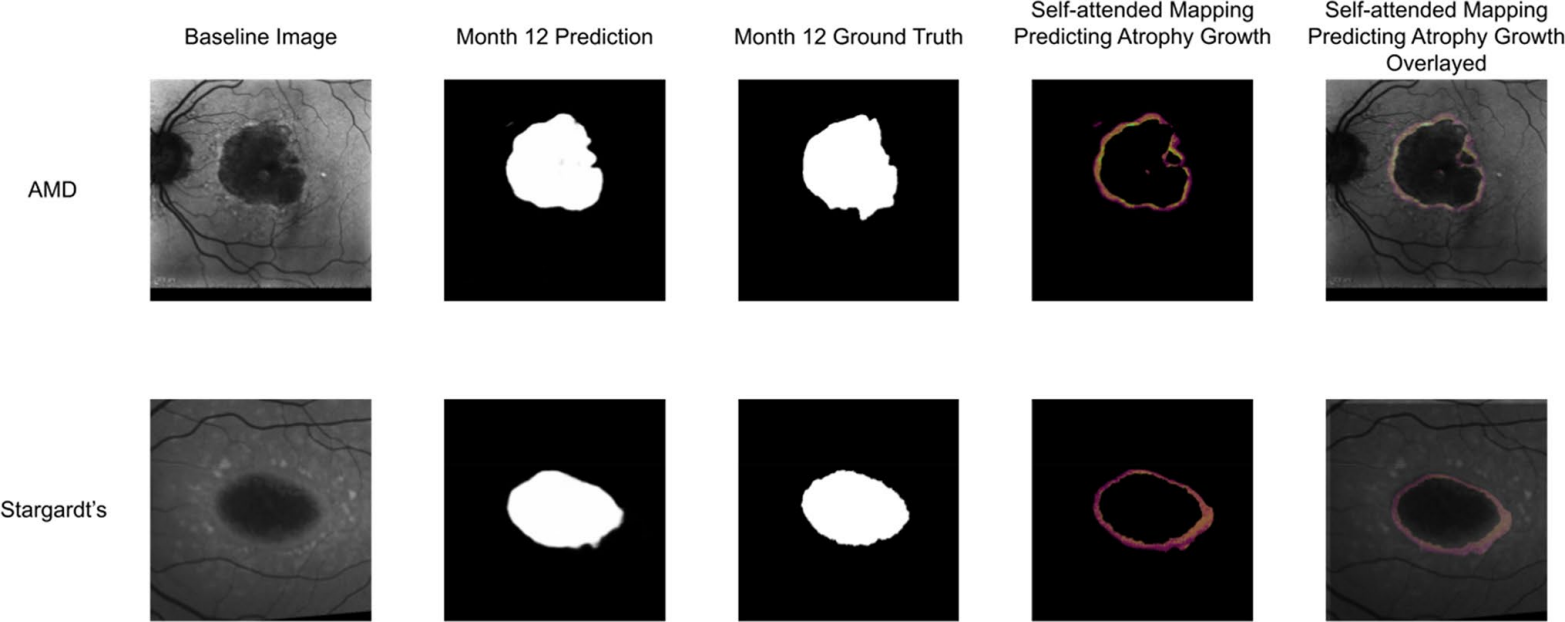
Illustration of the atrophy progression prediction results based on baseline images and Month 12 ground truth.

The performance results of the five measures for the automated segmentation and prediction of atrophic lesions with AMD and Stargardt are provided in Table 1. The percent improvements and Mann–Whitney test results obtained from MedCalc statistical software for each of the five measured results are compiled in Table 2. The 95% confidence intervals were obtained from Excel confidence interval function under descriptive statistics of data analysis. They measure the percent improvement of the self-attended U-Net over the regular U-Net on baseline AMD images and segmentation, the self-attended U-Net over the regular U-Net on baseline Stargardt images and segmentation, the self-attended U-Net on baseline AMD images and segmentation over Stargardt images and segmentation, and the self-attended U-Net on baseline AMD images and Month 12 segmentation over Stargardt baseline images and Month 12 segmentation. Figure 4 provides several sets of atrophy segmentation results of self-attended U-Net and the regular U-Net on baseline images with a general performance. Figure 5 illustrates the reconstruction and self-attended maps based on the baseline atrophy segmentation. Figure 6 illustrates the atrophy progression prediction results based on baseline images and Month 12 ground truth. Furthermore, distribution visualizations with histogram graphs for all the parameters in Table 1 are provided in supplementary materials 1 to 3. Data comparison visualizations with box-and-whisker graphs for all the comparisons in Table 2 are provided in supplementary materials 4 and 5.

**Table 1:** Performance results of automated segmentation and prediction of atrophic lesions for eyes with AMD and Stargardt with 95% CIs.

CNN	Disease	Visit	Dice	IoU	Accuracy	Sensitivity	Specificity
**AMD**							
U-Net	AMD	Month0	0.77 ± 0.05	0.66 ± 0.05	0.96 ± 0.01	0.78 ± 0.05	0.98 ± 0.01
Self-attended U-Net	AMD	Month0	0.85 ± 0.04	0.77 ± 0.05	0.98 ± 0.00	0.85 ± 0.04	0.99 ± 0.00
Self-attended U-Net	AMD	Month12	0.78 ± 0.05	0.68 ± 0.05	0.95 ± 0.01	0.75 ± 0.05	0.98 ± 0.00
**Stargardt**							
U-Net	Stargardt	Month0	0.65 ± 0.03	0.52 ± 0.03	0.90 ± 0.01	0.54 ± 0.04	0.99 ± 0.00
Self-attended U-Net	Stargardt	Month0	0.79 ± 0.03	0.69 ± 0.03	0.95 ± 0.01	0.73 ± 0.03	0.99 ± 0.00
Self-attended U-Net	Stargardt	Month12	0.76 ± 0.04	0.64 ± 0.04	0.94 ± 0.02	0.68 ± 0.04	0.99 ± 0.01

**Table 2:** Percent differences and Mann–Whitney test results between different CNNs for different diseases.

CNN 1 vs CNN 2	Disease 1 vs disease 2	Visit	Dice (*p-*value)	IoU (*p-*value)	Accuracy (*p-*value)	Sensitivity (*p-*value)	Specificity (*p-*value)
U-Net vs self-attended U-Net	AMD vs AMD	Month0	10.6% ± 1.3% (*p* < 0.0001)	17% ± 0.0% (*p* < 0.0001)	2.1% ± 0.0% (*p* < 0.0001)	9.4% ± 1.3% (*p* = 0.0011)	1.2% ± 1.0% (*p* = 0.2855)
U-Net vs self-attended U-Net	Stargardt vs Stargardt	Month0	22.0% ± 0.0% (*p* < 0.0001)	32% ± 0.0% (*p* < 0.0001)	5.4% ± 0.0% (*p* < 0.0001)	35.5% ± 1.9% (*p* < 0.0001)	−0.1% ± 0.0% (*p* < 0.0001)
Self-attended U-Net vs self-attended U-Net	Stargardt vs AMD	Month0	7.3% ± 1.3% (*p* < 0.0001)	13% ± 2.9% (*p* < 0.0001)	2.9% ± 1.0% (*p* < 0.0001)	16.6% ± 1.4% (*p* < 0.0001)	−0.2% ± 0.0% (*p* < 0.0001)
Self-attended U-Net vs self-attended U-Net	Stargardt vs AMD	Month12	1.9% ± 1.3% (*p* = 0.0067)	6% ± 1.6% (*p* = 0.0067)	1.4% ± 1.1% (*p* = 0.6585)	10.2% ± 1.5% (*p* = 0.0001)	−0.6% ± 1.0% (*p* < 0.0001)

Percent difference defined as *(CNN2 − CNN1)/CNN1*.

Mann–Whitney test results are indicated by the *p*-values.

## Discussion and conclusions

In this paper, we reported an automated system for the segmentation and progression prediction of AMD GA lesions and Stargardt atrophic lesions. The automated system utilizes a self-attended neural network which embeds the trainable attention mechanism into a fully convolutional neural networks, the U-Net, to focus “attention” on the notable image features for more efficient performance. The neural network generates soft-labels, which allows for more informative probabilistic predictions than deterministic hard labels. Such soft-labeled self-attended CNN system can efficiently highlight the important foreground information with an enhanced algorithm performance compared to a regular U-Net and does not need to use complex external training components.

Based on the quantitative performance evaluation results as shown in Tables 1 and 2, we can see that the self-attended U-Net had better performance than the regular U-Net almost across the board, having a 2.1% higher accuracy performance on baseline FAF images for AMD GA segmentation and a 5.4% higher accuracy on baseline FAF images for Stargardt atrophy segmentation. This is along with a 10.6% higher Dice coefficient and 17% higher IoU for AMD GA segmentation, and a 22% higher Dice coefficient and a 32% higher IoU for Stargardt atrophy segmentation. Overall, the self-attended U-Net had an accuracy of 0.98 for baseline AMD GA segmentation, 0.95 for predicting AMD atrophy progression over 12 months, 0.95 in baseline Stargardt atrophy segmentation and 0.94 for predicting Stargardt atrophy progression over 12 months. The higher sensitivity in the self-attended U-Net’s performances indicate that it is much better in recognizing true positive regions of atrophy when compared to the regular U-Net. In regard to the performance for the two different diseases, the self-attended U-Net performed generally better in automatically segmenting AMD GA lesions when compared to segmenting Stargardt atrophic lesions. This is likely due to a difference in the typical atrophy morphology in the two diseases. While atrophic AMD GA is well-demarcated and generally uniform in its hypoautofluorescence on FAF imaging, Stargardt atrophy is frequently less well-demarcated and less uniform^5,6^. The specificity results were also extremely high in each model, with a specificity of 0.99 for all tests except for AMD self-attended U-Net Month 12 and the baseline regular U-Net, which both had specificities of 0.98. This is likely due, in part, to the large amount of non-atrophy in the original images, meaning that false positives account for a relatively small portion of all regions of non-atrophy. Certain background-regions of non-atrophy are also much easier to detect, due to their distinct differences from regions of atrophy.

Several visual results of the AMD GA and Stargardt atrophy segmentation and progression prediction with the self-attended U-Net and the regular U-Net are provided in Figs. 4, 5 and 6 with an overall average level of performance. Overall, the self-attended U-Net yields better performance than the regular U-Net, particularly for the multi-focal pattern lesions. As shown in the second and third rows in Fig. 4, the regular U-Net may present ‘under-segmentation’ if the multi-focal lesions have uneven image intensity distribution (the second row in Fig. 4) and may present ‘over-segmentation’ if a non-atrophy region (e.g., the optic nerve head region in the FAF image on the third row in Fig. 4) has similar intensity level with the atrophy regions. The feature mapping results of the self-attended U-Net and the regular U-Net are shown in Fig. 5. In the process of baseline atrophic lesions’ segmentation, the activation heatmaps displayed from our self-attended neural network tend to focus on the notable features of the foreground. Because the self-attended mechanism directly involves in the training of the neural network, it urges the activations to concentratedly attend on the markedly reduced hypo-fluorescence regions interested (i.e., atrophic regions with complete RPE and corresponding fluorescence loss). The reconstruction heatmaps in the process of U-Net baseline atrophic lesions’ segmentation tend to have expanded feature regions than the real atrophy regions, because the reconstruction heatmaps are based on the post-doc activations of the regular U-Net, which do not utilize such an attention mechanism. Furthermore, as shown in Fig. 6, the heatmaps with the self-attended mechanism can capture the feature rings (i.e., the intensity changes due to the early damages of the retina) which correspond with the atrophy growth after 12 months. As shown on the baseline FAF images, around the borders of markedly reduced hypo-fluorescence atrophic regions, scattered autofluorescence reductions have occurred. It is indicative of the incomplete loss of the RPE and corresponding fluorescence and an early sign of continuous growth of atrophic lesions in the future. The self-attended activation heatmaps can capture such early signs of future disease growth with the CNN trained on the baseline incomplete loss of fluorescence and follow-up ground truth.

While this study demonstrates the advantages/improvements in segmentation and progression prediction, it also has some drawbacks. (1) The nature of the deep learning algorithms requires larger training datasets for optimal algorithm performance. While the fully convolutional neural networks used in this study work well with smaller datasets, larger datasets are preferred to achieve optimal performance. Our datasets are relatively small as we required patients to have longitudinal visits. (2) AMD and especially Stargardt progression are generally slow. The growth of the atrophic regions for both AMD and Stargardt disease within 12 months are not distinct as shown in Fig. 3. Also, because the FAF images are 2D projections of the 3D retina, the specific retinal layers impacted by the disease process are not clearly defined on the 2D FAF images, and this may limit our ability to extract biomarkers predictive of disease progression. Future studies with larger 3D image datasets with longer longitudinal follow-up will be important to further improve algorithm performance and identify early biomarkers which can predict progression. Also, these models can only be used when GA or Stargardt’s atrophy is predetermined to be present within the image.

In summary, in this paper, we reported a deep learning system using a self-attended neural network for the automated segmentation and progression prediction of AMD GA lesions and Stargardt atrophic lesions. Compared with the start-of-the-art regular U-Net, the self-attended neural network demonstrates a consistent enhancement of performance for both AMD and Stargardt diseases. The developed self-attended mechanism can be applied on the visual discovery of early AMD features based on longitudinal image data with longer visit times.

## Supplementary Information

Supplementary Information 1.

Supplementary Information 2.

Supplementary Information 3.

Supplementary Information 4.

Supplementary Information 5.

## Data Availability

The datasets generated and/or analysed during the current study are not publicly available due to the patients’ privacy and the violation of informed consent but are available from the corresponding author on reasonable request.

## Code availability

The code generated during the study is accessible from the corresponding author based on reasonable request and subject to the rule/regulatory of the institute.
