# Supplementary material for: Automated segmentation and feature discovery of age-related macular degeneration and Stargardt disease via self-attended neural networks
Source: Sci Rep. 2022 Aug 26;12:14565. doi: 10.1038/s41598-022-18785-6 (PMC9418226; doi:10.1038/s41598-022-18785-6)
Supplement: Supplementary file 1 — Supplementary Information 1. [file 41598_2022_18785_MOESM1_ESM.docx]

| 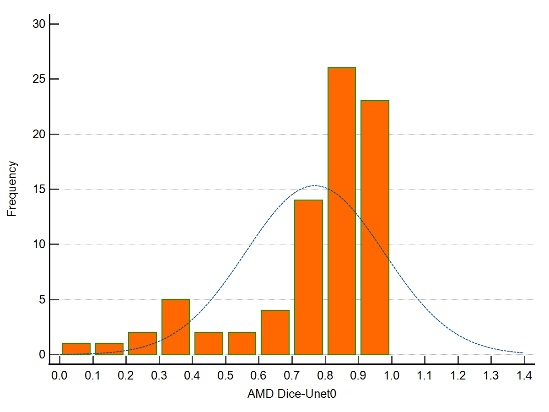 | 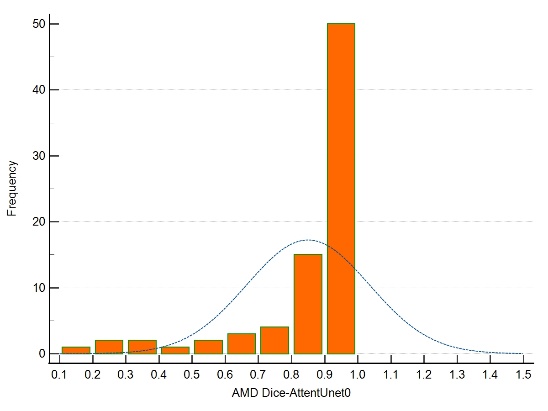 |
| --- | --- |
| 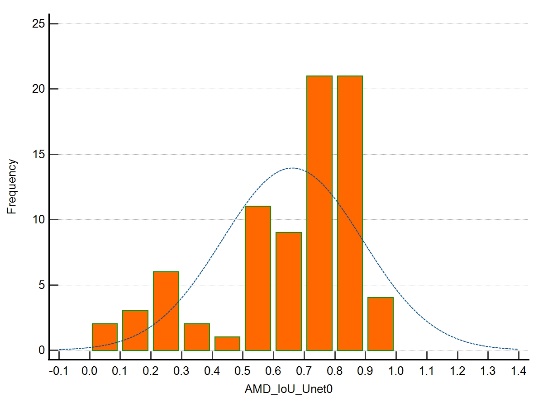 | 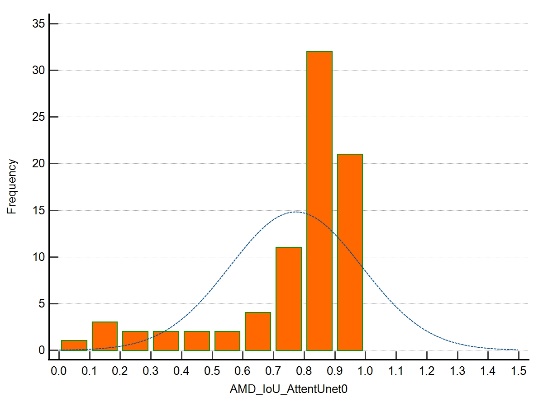 |
| 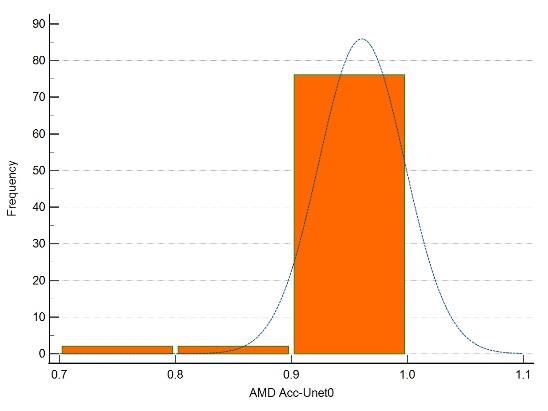 | 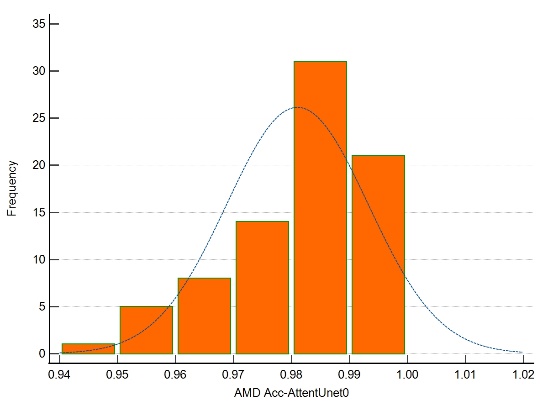 |
| 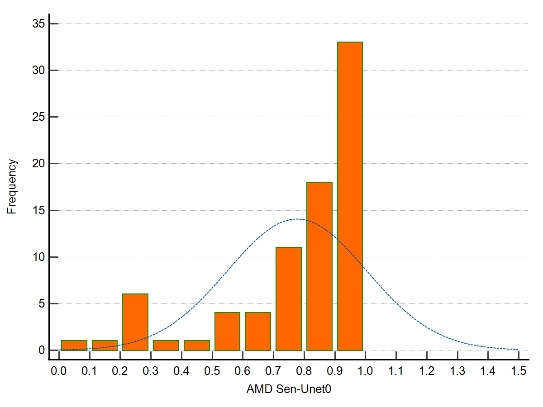 | 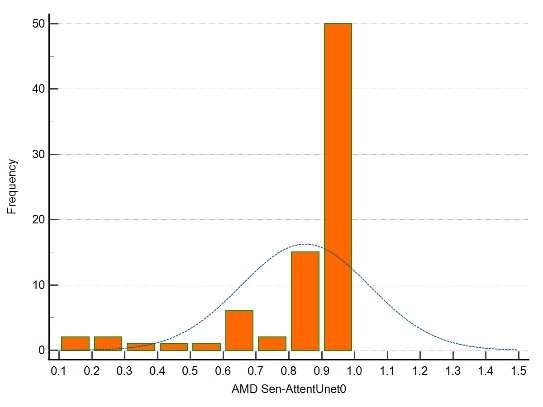 |
| 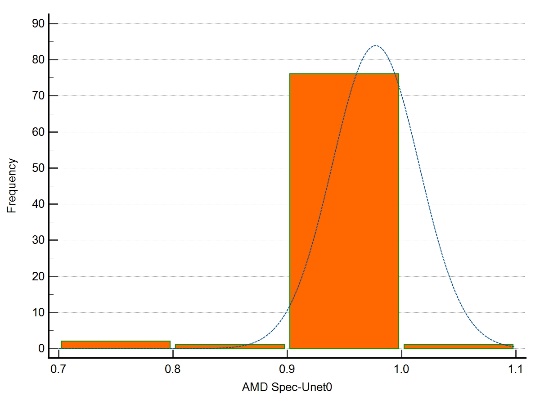 | 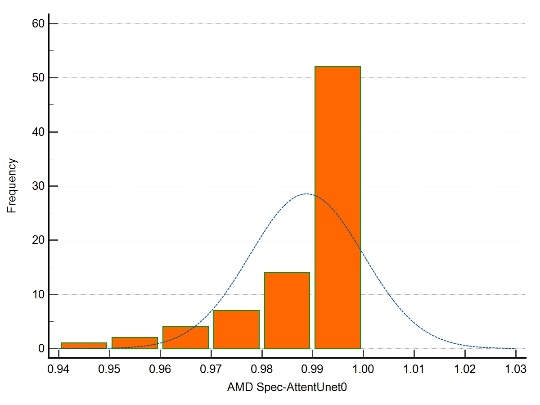 |

Supplement 1: Histogram distributions of Unet and Self-attended Unet results for AMD data at Month0 as shown in Table 1.
