# Supplementary material for: Automated segmentation and feature discovery of age-related macular degeneration and Stargardt disease via self-attended neural networks
Source: Sci Rep. 2022 Aug 26;12:14565. doi: 10.1038/s41598-022-18785-6 (PMC9418226; doi:10.1038/s41598-022-18785-6)
Supplement: Supplementary file 4 — Supplementary Information 4. [file 41598_2022_18785_MOESM4_ESM.docx]

| Unet and Self-attended U-net for AMD | Unet and Self-attended U-net for Stargardt |
| --- | --- |
| 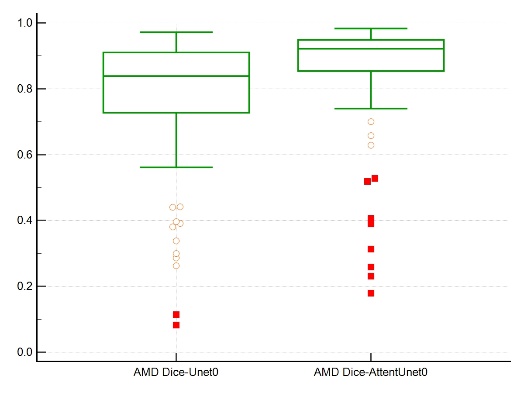 | 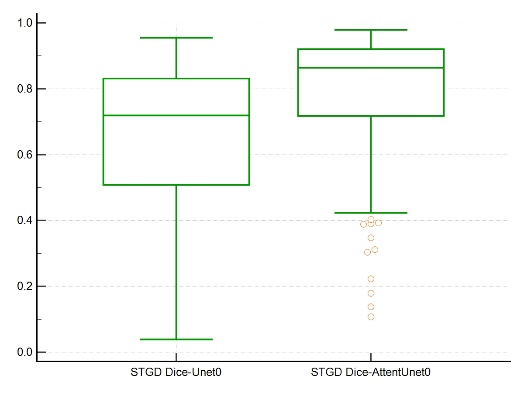 |
| 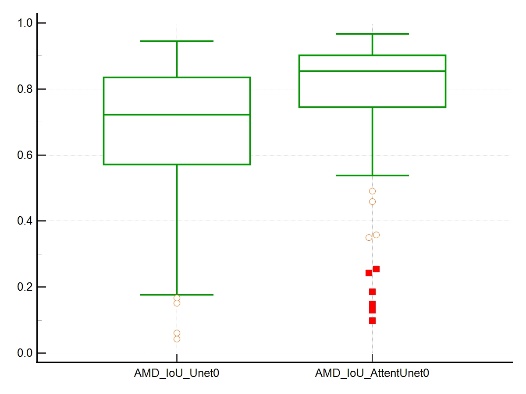 | 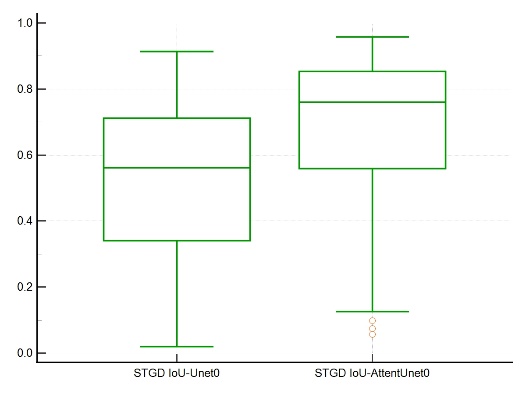 |
| 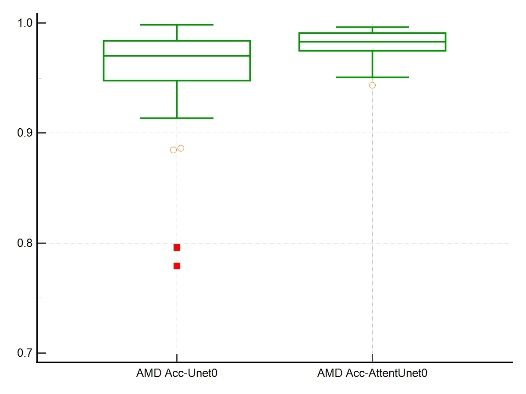 | 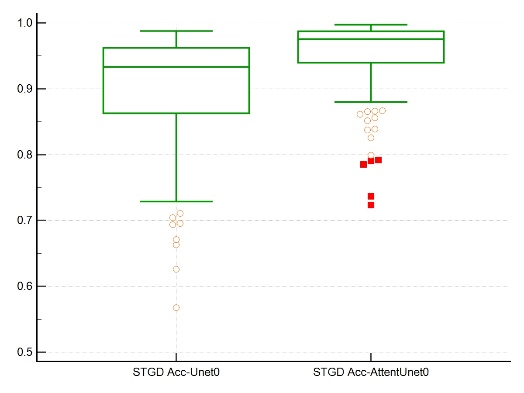 |
| 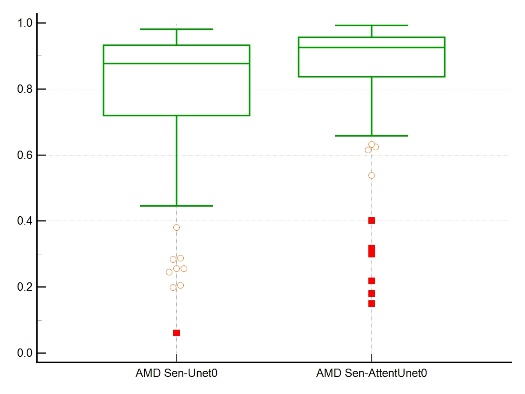 | 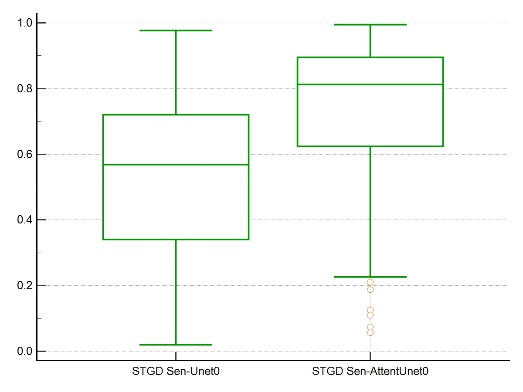 |
| 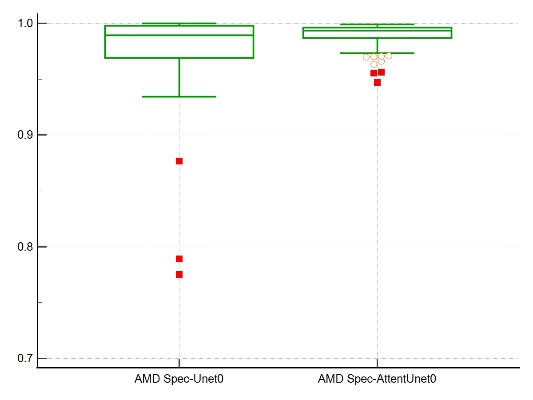 | 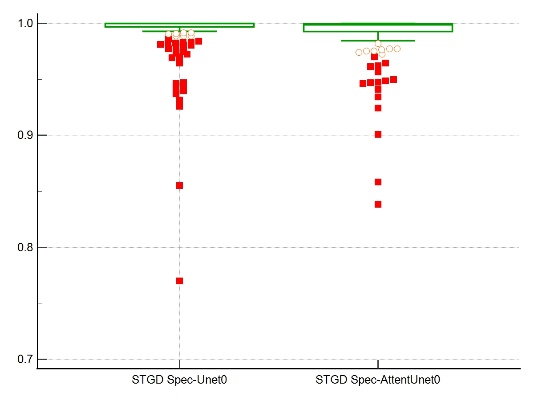 |

Supplement 4: Box-and-Whisker plots for comparisons of Unet and self-attended-Unet results for both AMD and Stargardt data as shown in Table 2. STGD: Stargardt.
