# Supplementary material for: Automated segmentation and feature discovery of age-related macular degeneration and Stargardt disease via self-attended neural networks
Source: Sci Rep. 2022 Aug 26;12:14565. doi: 10.1038/s41598-022-18785-6 (PMC9418226; doi:10.1038/s41598-022-18785-6)
Supplement: Supplementary file 5 — Supplementary Information 5. [file 41598_2022_18785_MOESM5_ESM.docx]

| Month 0 | Month12 |
| --- | --- |
| 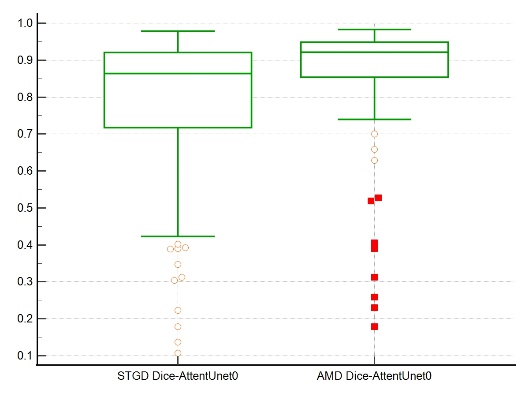 | 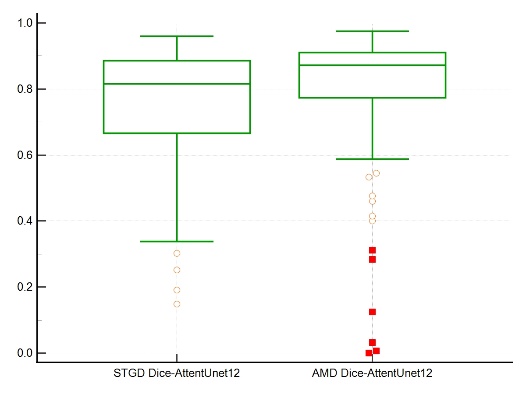 |
| 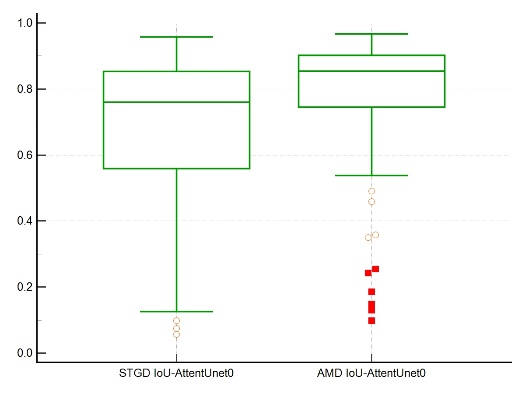 | 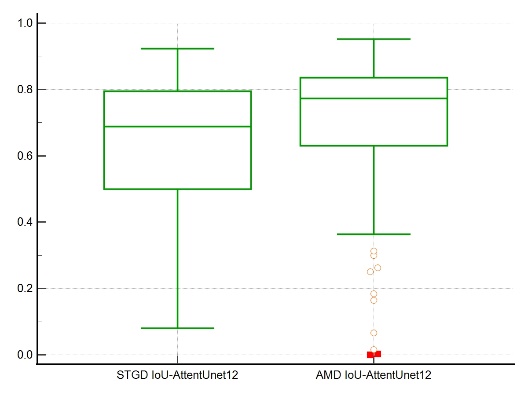 |
| 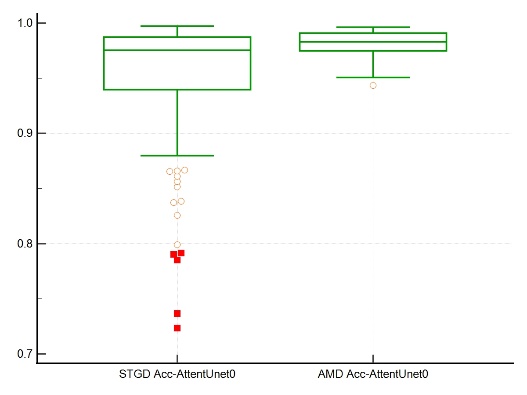 | 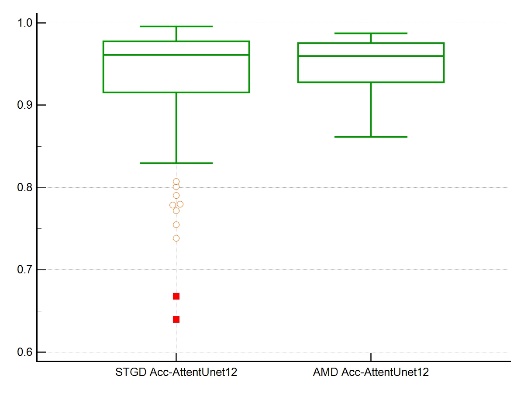 |
| 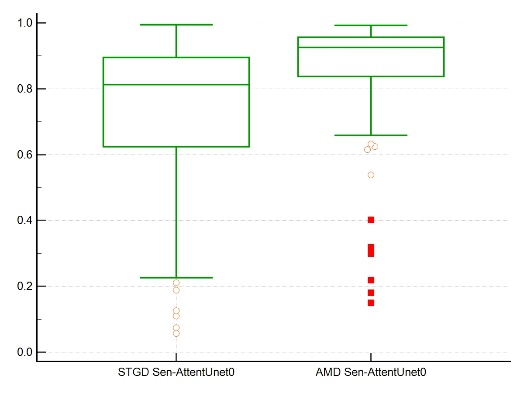 | 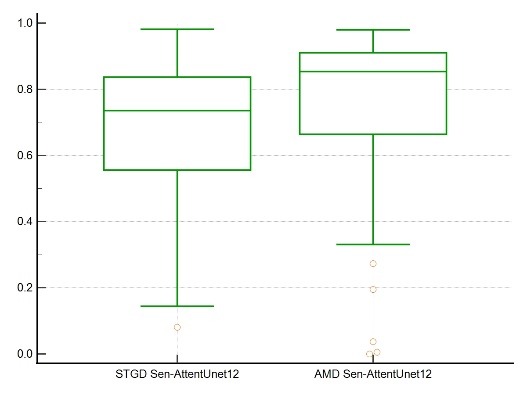 |
| 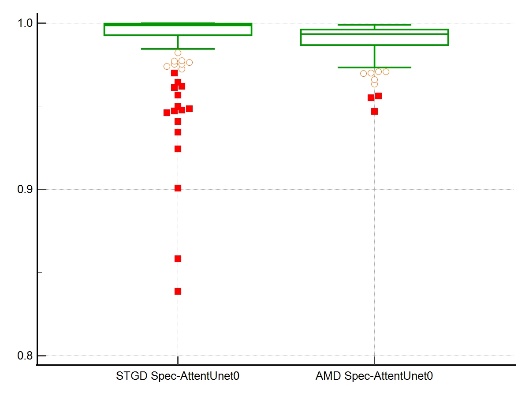 | 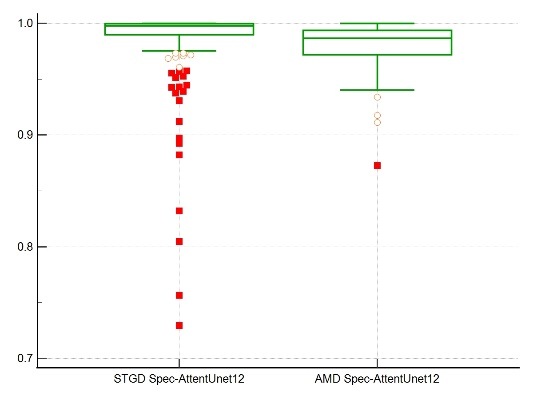 |

Supplement 5: Box-and-Whisker plots for comparisons of self-attended-Unet results between AMD and Stargardt data at both Month0 and 12 as shown in Table 2. STGD: Stargardt.
